# Wi-Fi Access Point Design Concept Targeting Indoor Positioning for Smartphones and IoT

**DOI:** 10.3390/s22030797

**Published:** 2022-01-21

**Authors:** Mohamed S. El-Gendy, Imran Ashraf, Samy El-Hennawey

**Affiliations:** 1 Microstrip Circuits, Electronics Research Institute, Cairo 11843, Egypt; mohamadalgendy2004@gmail.com; 2 Department of Information and Communication Engineering, Yeungnam University, Gyeongsan 38541, Korea; 3 Electronics and Communications Engineering Department, Misr International University, Cairo 11828, Egypt; mohamed.elhennawey@miuegypt.edu.eg

**Keywords:** indoor positioning systems, wireless networks, IoT, phased antenna arrays

## Abstract

Indoor positioning systems (IPS) have been regarded as essential for many applications, particularly for smartphones, during the past decade. With the internet of things (IoT), and especially device-to-device (D2D) cases, the client is supposed to have a very simple structure and low cost. It is also desirable that the client contains minimal software modules specifically for IPS purposes. This study proposes a new IPS technique that satisfies these conditions. The evaluation of the technique was previously executed based on a manual procedure. This technique utilizes Wi-Fi technology in addition to a new design of two orthogonal phased antenna arrays. This paper provides a complete design of a Wi-Fi access point (AP), considered as the proof of concept of a commercial AP. For the system to be fully automatic, the proposed architecture is based on a Raspberry Pi, external Wi-Fi modules, a powered universal serial bus (USB) hub, and two orthogonal phased antenna arrays. The phases of each antenna array are governed by extra-phase circuits as well as a radio frequency (RF) switch. Extensive design parameters have been chosen through parametric sweeps that satisfy the design conditions. Software testing results for the antenna arrays are included in this paper to show the feasibility and suitability of the proposed antenna array for IPS.

## 1. Introduction

Positioning and localization have gained growing interest during the past decade primarily due to the wide proliferation of mobile devices, the major part of which is the smartphone. The inclusion of rich embedded sensors in the smartphone such as the global positioning system (GPS) chip and, more recently, navigation satellite system (GNSS) chip, accelerometer, Wi-Fi module, gyroscope, and magnetometer, etc. have made smartphone-based positioning a potential consumer industry. For outdoor positioning, GNSS based solutions serve as the de facto technology and provides a meter level accuracy [1]. As GNSS based solutions work only in outdoor situations (indoors, it has limitations of accuracy), several systems have been developed for indoor scenarios [2,3,4,5] and the term indoor positioning systems (IPS) has become widely known. There are several IPS techniques with a variety of accuracy achieved, the type of signaling used, as well as the system complexity, etc. [6,7,8,9]. Therefore, for each application, the system designer is to choose the technique that is suitable for the application. In internet of things (IoT) industrial applications, particularly for its new generation [10], the client is required to have a very simple architecture and low cost. This is particularly important for the case of device-to-device (D2D) scenarios, as well as in smartphones. In addition, some IoT applications mandate certain levels of position accuracy. This implies that for D2D communication, simplicity and accuracy are both needed.

For providing accurate indoor position for smartphone-based location-based services (LBS), several technologies have been investigated, such as Bluetooth low energy [11], pedestrian dead reckoning [12], magnetic field-based positioning [13,14], and Wi-Fi [5], to name a few. Each of these technologies has its own merits and demerits regarding the positioning environment, infrastructure, accuracy, and delay time, etc. Despite its limitation of signal blocking, interference, attenuation, and shadowing problems, Wi-Fi-based IPS have been widely researched and adopted [15]. However, such approaches are intended for existing Wi-Fi access points (APs) that are widely deployed in indoor environments and limited by energy usage. Specifically, such approaches are not suitable for modern IoT devices and device-to-device communications. Recently, a new method, based on orthogonal antenna arrays in Wi-Fi has been proposed [16]. The basic idea is to divide the indoor hall into rows and columns (*X* and *Y*). The footprint of each row ($X_{i}$) and column ($Y_{i}$) is spotted by the two orthogonal phased antenna arrays in a swept manner. Upon spotting a certain row or column, the Wi-Fi access point (AP) is to transmit a distinct service set identifier (SSID) with the name of the row ($X_{i}$) or the column ($Y_{i}$). As such, the Wi-Fi receiver receives two distinct SSIDs indicating its position right away without any additional overhead required. The proposed IPS technique [16] has been evaluated in [17]. However, the evaluation is based on using an off-the-shelf AP while manually changing the antenna array phases. The field of study would certainly benefit from an evaluation investigating an automatic operation.

Despite the available multitude of Wi-Fi-based indoor positioning approaches, the provided localization accuracy does not meet the standards for providing the high quality of service for LBS. In addition, these APs are limited by energy usage, latency, and other similar factors. Consequently, several approaches and novel methods have been presented. For example, ref. [18] proposes MapFi to build autonomous Wi-Fi maps for heterogeneous indoor environments without site survey. A generic method is presented to estimate the angle of arrival (AoA) which assists the map building module. Wi-Fi APs are segmented into different groups with respect to their channel state information (CSI) and merged to generate a global map. Results indicate significant improvement for tail localization error. For increasing the localization accuracy, sensor fusion is an attractive choice where the data from multiple sensors of a smartphone are utilized. For example, ref. [19] integrates the Wi-Fi data along with and inertial sensors such as gyroscopes and accelerometers for enhancing the location accuracy. Initially, the proximity is measured using the received signal strength (RSS) measurement between the smartphone and Wi-Fi APs. Later, proximity is combined with PDR using an extended Kalman filter for more accurate location estimation. Another potential solution is the optimal placement of the Wi-Fi APs to ensure the proper coverage for all the areas intended for localization. As the Wi-Fi APs are deployed for communication, instead of the localization perspective, their deployment is not ideal for localization. Alsmady et al. provide a genetic algorithm-based approach in [20] to estimate the optimized deployment of Wi-Fi APs to increase localization accuracy. Simulation results indicate that the uniqueness of Wi-Fi fingerprints can be increased up to 90% using the proposed approach. Besides providing the IPS approaches for increased accuracy, energy efficiency, resilience, and latency are not sufficiently studied in the existing literature.

The rest of the paper is structured as follows. In the following Section 2, the background material is presented, including the technique in [16], as well as the manual evaluation previously made. Section 3 presents the details of the new design of the antenna arrays. Section 4 provides the overall system assembly. Discussions and future perspectives are presented in Section 5. Finally, the paper is concluded in Section 6.

## 2. Background and Related Material

The current era is marked by the wide proliferation of the smartphone which contains a rich variety of embedded sensors such as an accelerometer, barometer, lux meter, Wi-Fi, and magnetic field sensor. The inclusion of these microelectromechanical sensors (MEMS) opened and set new directions for the consumer industry, e.g., phone marketing, LBS, and on-the-go services, etc. For all these and other, similar service industries, user location serves as the backbone on which the service industry is established. The accurate and precise location is directly linked to the higher quality of service and customer satisfaction. Modern smartphones such as the Samsung Galaxy S11 and iPhone 12 provide 5G Wi-Fi gigabit transceiver and support IEEE 802.11 Wi-Fi family, including 802.11aj for mmWave. Implementation of 3-stream 802.11ac specification enables these smartphones to achieve higher data rates of up to several gigabits per second. The Smartphone Wi-Fi chip has a dual-band transceiver with a 256-QAM modulation scheme to increase the data transfer efficiency. Utilizing the Wi-Fi signals from indoor deployed Wi-Fi APs and smartphone embedded chips, indoor localization can be performed using the received signal strength (RSS) of these APs that have unique basic service support identification (BSSID).

**Figure 1:**
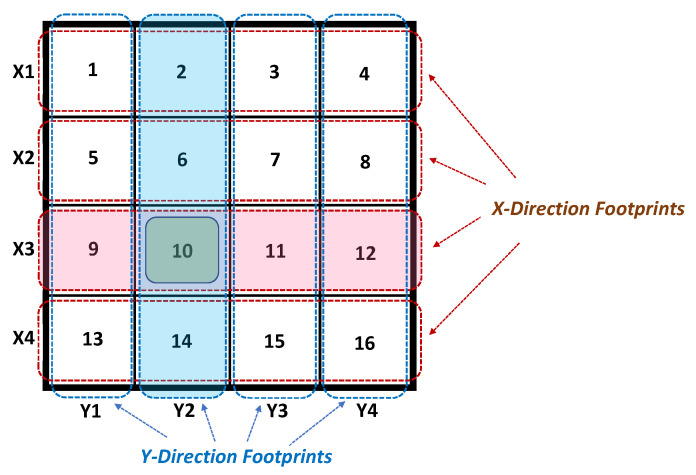
Indoor hall layout and footprints.

The new proposed IPS method [16] is reviewed here and its evaluation process is summarized [17]. The main idea is to divide the indoor hall floor into rows, denoted by Xi, and columns, denoted by Yi. The Wi-Fi antenna consists of two orthogonal phased antenna arrays. The first antenna array produces a spot beam or footprint of near elliptic shape, covering one row at a time. The other antenna array produces a spot beam or footprint, also of near elliptic shape, covering one column at a time. The two antenna arrays are swept in their phases sequentially to cover all rows and all columns, thus covering the entire indoor hall. During spotting a row or a column, the AP produces a distinct service set identifier (SSID) or simply ID, corresponding to the specific row or column it covers, i.e., Xi or Yi. Then, the Wi-Fi receiver at a certain square (intersection of a certain row and a certain column) will receive two IDs, probably not at the same instant of time, but close during the sweep cycle. This is shown in Figure 1, assuming a hall of 4 × 4 m${}^{2}$, divided into four rows and four columns, i.e., each square is of 1 m side length. The choice of the number of rows and columns depends on the hall layout, as well as the desired accuracy.

In Figure 1, the instance is showing that the antenna arrays are phased to cover the third row and the second column, i.e., covering square #10. A Wi-Fi receiver in square #10 receives two SSIDs: X3 and Y2. Therefore, the receiver receives X3 (row) and Y2 (column) and consequently determines its coordinates in the hall as (X3,Y2). The evaluation of the above method has been carried out in [17]. The design of the antenna is based on two-phased antenna arrays with four antenna elements. The phases of the antenna array are obtained through an extra feed circuit for each row or column. The extra phase circuit produces phases for one of the antenna arrays to cover a certain row or column. Obviously, there are four different extra phase circuits to cover the entire four rows or the four columns.

The evaluation operation [17] is executed with an off-the-shelf access point, where the SSID is set manually. In each step, only one antenna array is operational with one specific extra phase circuit associated with a certain row (or column) and the corresponding SSID. Measurement is then made throughout the hall to verify that only the spotted row (or column) receives the corresponding SSID (Xi or Yi). It is obvious that this manual operation is exhausting, and this is the motivation of this paper. In the following sections, the new antenna design and other considerations are presented.

## 3. Proposed Antenna Array Assembly Design

This study presents a complete design of the IPS system based on the technique in [16] and a continuation of the evaluation started in [17] is presented. The complete AP system design is completed and it is comprised of

- a microcomputer system, Raspberry Pi 4;
- a powered universal serial bus (USB) hub;
- two external Wi-Fi modules;
- the switchable antenna assemblies.

The phase sweeping process is performed through the use of extra-feed circuits switched by two radio frequency (RF) switches, controlled by the Raspberry Pi.

The antenna assembly is based on the 5 GHz band to reduce its size. Each antenna assembly consists of an antenna array, power divider circuit, extra-phase circuits, and subminiature version A (SMA) adaptors. To obtain two orthogonal beams (each has an ellipse shape) two perpendicular linear microstrip antenna arrays with sided cages are designed. The footprint (i.e., projection of the radiated beam) is also an ellipse shape. Two perpendicular footprint ellipses are utilized to cover one horizontal ellipse cell (i.e., row) and one vertical ellipse cell (i.e., column). The whole size of the serving zone to be covered is 4 m by 4 m, as shown in Figure 1.

Keeping in view the remarkable merits of microstrip technology such as light weight, small area, small thickness profile, and low implementation cost; the design of the antenna array, feeding circuit, and the extra-phase circuits are implemented using that technology. Based on that, the proposed RF system (i.e., antenna array with its feeding circuit) is advantageous to be mounted on either the wall or the ceiling. Furthermore, the microstrip technology can be easily integrated with the other microwave integrated circuits. Therefore, each proposed antenna array and its feeding circuit network are connected to the other part of the AP circuit using a 50 Ω coaxial cable with a U.FL connector. The proposed antenna array and its feeding networks are designed based on the IEEE 802.11 ac Wi-Fi standard at the operating frequency of 5 GHz for size reduction.

The first step is to design a single antenna element of the array. The rectangular printed patch antenna is selected to be the radiator element because it provides a radiation pattern at broadside direction (i.e., perpendicular on the surface of the antenna). The proposed RF system (i.e., radiator and feeding circuit) is also designed at the same operating frequency of 5 GHz. The selected AP operates at the spectrum frequency range between 5.15 GHz and 5.35 GHz with the center frequency of 5.25 GHz. The second step is designing the antenna array. As the required footprint shape is an ellipse shape, a linear antenna array is chosen. The number of the antenna elements for the linear array is selected to be four, and this number can be increased or decreased according to the cell size, i.e., the required accuracy. The space between the antenna element should be selected carefully in order to prevent the presence of the grating lobes. Therefore, a simulation sweep is performed to find an optimized separation value. The third step is to design the feeding circuit that excites the proposed linear antenna array. The feeding circuit board consists of one T-shaped power divider circuit, with equal power division ratio and equal phases at the end of its output ports and two units of Wilkinson power dividers with equal power division ratio and equal phases at the end of their output ports. The final step is to design an extra-phase circuit board that is connected at the output of the power divider circuit for the two orthogonal antenna arrays. The main function behind these extra-phase circuits is to calculate different values of phases at the input of the linear antenna array. Different values are used to result in an inclined beam with an inclined angle (Θ) to cover other rows or columns that require different inclined radiation beams to be covered.

### 3.1. Antenna Element: Rectangular Patch Antenna Design

**Figure 2:**
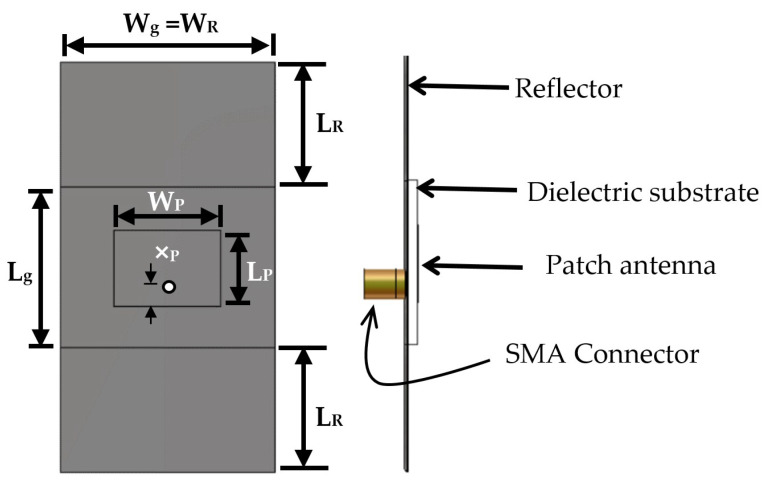
Rectagular microstrip antenna with its reflector.

The first step is to design and simulate a single antenna element. A rectangular microstrip patch antenna is designed on a dielectric substrate of FR-4 with relative permittivity $\epsilon_{r}$ = 4.5, substrate thickness of h = 1.5 mm, and loss tangent of 0.025, as shown in Figure 2. The patch length ($L_{p}$), width ($W_{p}$), and the feeding position ($X_{p}$) are calculated using the following Equations [21]

(1) $$\begin{array}{c} L_{p}=\frac{\lambda_{g}}{2}-2\Delta L \end{array}$$

(2) $$\begin{array}{c} W_{p}=\frac{C}{2f_{0}}\sqrt{\frac{2}{\epsilon_{r}+1}} \end{array}$$

(3) $$\begin{array}{c} Re\left[Z_{in}\left(\mathrm{at}\;50\Omega \;\mathrm{location}\right)\right]=Re\left[Z_{in}\left(\mathrm{at}\;\mathrm{patch}\;\mathrm{edge}\right)\right]cos^{2}\left(\frac{\pi }{L_{P}}X_{p}\right)] \end{array}$$

where,

(4) $$\begin{array}{c} \lambda_{g}=\frac{C}{f_{0}\sqrt{\epsilon_{eff}}} \end{array}$$

(5) $$\begin{array}{c} \varepsilon_{eff}=\frac{\epsilon_{r}+1}{2}+\frac{\epsilon_{1}-1}{2}{\left[1+12\frac{h}{W_{P}}\right]}^{-0.5} \end{array}$$

(6) $$\begin{array}{c} \Delta L=0.412h\frac{\left(\epsilon_{eff}+0.3\right)\left(\frac{W}{h}+0.264\right)}{\left(\epsilon_{eff}-0.258\right)\left(\frac{W}{h}+0.8\right)} \end{array}$$

where $\lambda_{g}$ is the wave guide wave length, ΔL is the incremental patch length due to fringing field phenomena, *C* is the speed of light (C=3×108 m/s), $f_{0}$ is the resonant center frequency ($f_{0}$ = 5.25 GHz), $\epsilon_{r}$ is the relative permittivity ($\epsilon_{r}$ = 4.5), $\epsilon_{eff}$ is the effective relative permittivity, and *h* is the substrate thickness.

**Figure 3:**
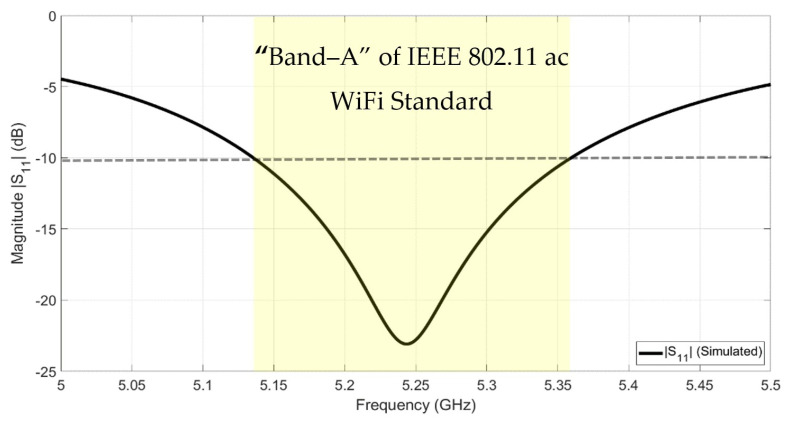
The reflection coefficient ($S_{11}$) of the rectangular patch antenna.

Table 1 shows the optimum dimension values for the designed rectangular antenna. The simulated reflection coefficient ($S_{11}$) of the antenna element is shown in Figure 3. The bandwidth (BW) of the designed patch antenna is from 5.15 GHz to 5.35 GHz which is qualified to be operated in the AP’s transmitting operation (Band-A) with maximum power 200 mW.

### 3.2. Linear Antenna Array Design: Ideal Excitation

**Figure 4:**
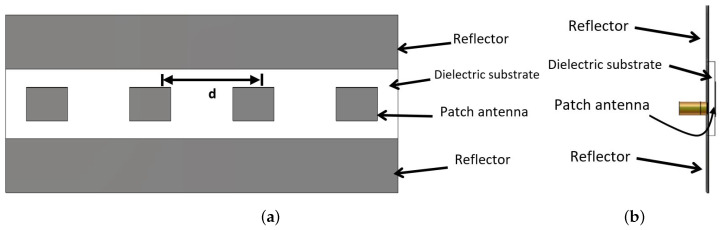
Rectangular microstrip linear antenna array with its reflector, (**a**) Elevation, and (**b**) Side view.

**Figure 5:**
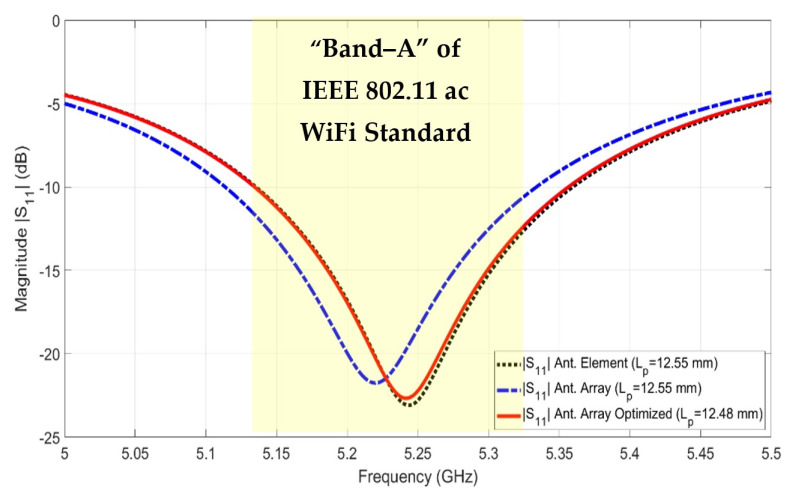
The effect of the mutual coupling on the reflection coefficients |$S_{nn}$|.

The second step is to design a linear array to achieve an ellipse footprint pattern. Four antenna elements are placed in the horizontal axis to obtain a vertical footprint with an ellipse shape, as shown in Figure 4. Another four antenna elements are placed in the vertical axis to obtain a horizontal footprint with an ellipse shape. Therefore, two orthogonal linear antenna arrays are designed. The separated distance (*d*) between each two antenna elements is selected to gain the optimum value. The optimum value is *d* = 43 mm (0.753 $\lambda_{0}$), which is in the range $\lambda_{0}/2<d<\lambda_{0}$. The patch length is re-optimized further after placing the antenna element in an array. This is because the mutual coupling between the antenna arrays affects the antenna performance (i.e., reflection coefficient |$S_{nn}$|: *n* = 1, 2, 3, and 4), as shown in Figure 5. It is depicted that the patch length is decreased from 12.55 mm to 12.48 mm to cover the AP’s Wi-Fi transmitting band (Band-A) which extends from 5.15 GHz to 5.35 GHz.

**Figure 6:**
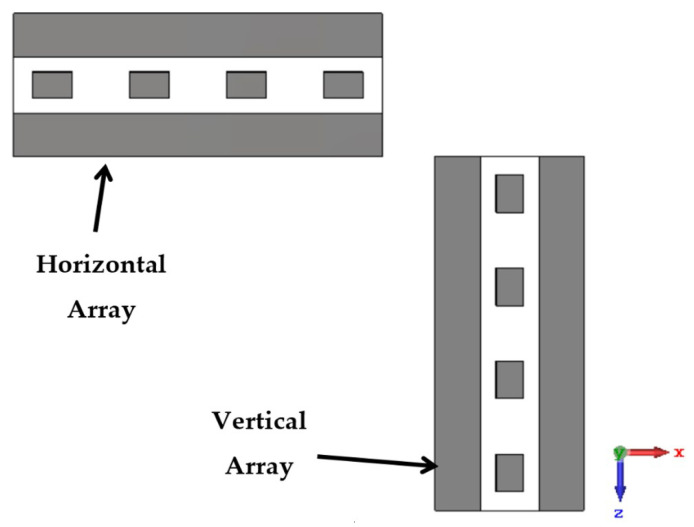
The two orthogonal antenna arrays.

The back radiation can be eliminated by extending the metallic ground plane in the Y-axis direction. Table 2 shows the gain values at different values of reflector length ($L_{R}$). It is shown in Table 2 that the optimum gain is 11.5 dBi at the reflector length of 10 mm. The array is designed on the same substrate material with which the antenna element is designed (i.e., FR-4 material). The size of the linear antenna array becomes 163.44 mm × 25.92 mm. Figure 6 shows the two orthogonal antenna arrays; one is located at the vertical view, while the other is located at the horizontal view to obtain the desired inverted letter of ’L’ design.

### 3.3. Design of Feeding Network and Extra-Phase Circuits

**Figure 7:**
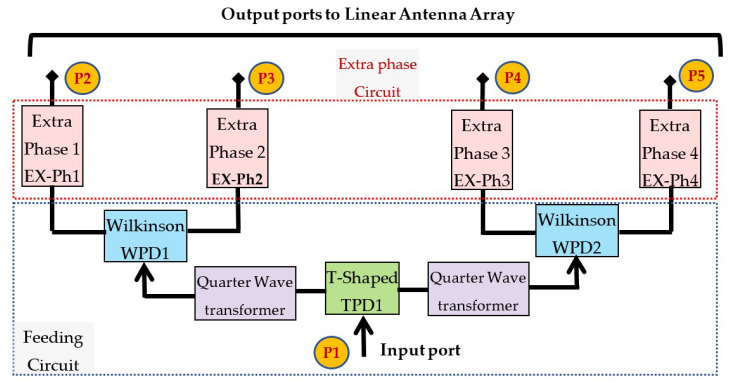
The schematic diagram of the proposed feeding circuit and extra-phase.

The third step is to design the feeding circuit with its extra-phase circuit that is used to excite the linear antenna array. Figure 7 shows the schematic diagram of the proposed feeding circuit and extra-phase. Figure 7 indicates that the feeding circuit consists of one T-shaped power divider circuit (TPD1) at the input of the feeding circuit and two Wilkinson power dividers (WPD1 and WPD2). All the T-shaped power dividers and the Wilkinson power dividers circuits are equal power division ratios and equal phases at the end of the output ports.

**Figure 8:**
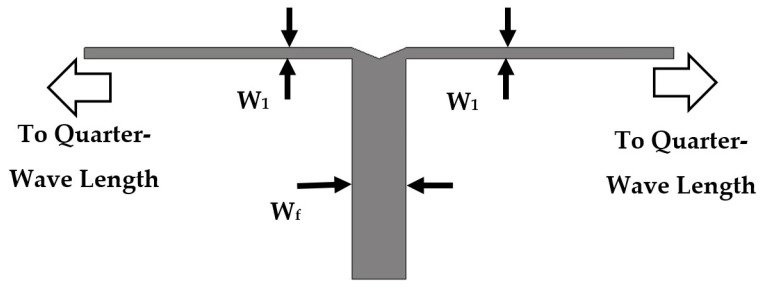
T-shaped power divider printed layout.

**Figure 9:**
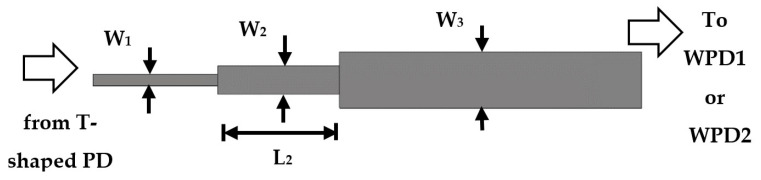
The designed quarter-wave transformer printed layout.

The feeding circuit consists of a T-shaped power divider, a quarter-wave transformer, and a Wilkinson power divider. The feeding network circuit is designed on FR-4 substrate material with $\epsilon_{r}$ = 4.5, thickness h = 1.5 mm, and loss tangent of 0.025. Figure 8 shows the printed layout of the T-shaped power divider. The input 50 Ω microstrip line is divided into two 100 Ω microstrip lines. The widths of the input/output impedances $Z_{F}$ = 50 Ω and $Z_{1}$ = 100 Ω transmission lines are $W_{F}$ = 2.78 mm and $W_{1}$ = 0.57 mm, respectively. In Figure 9, a quarter-wave transformer is utilized to match between the microstrip line width of $Z_{1}$ = 100 Ω and the microstrip line width of $Z_{3}$ = 50 Ω (i.e., input feed of Wilkinson power divider WPD1 or WPD2) using Equation 7 below [21]. Equation 2 is valid in the case that the length ($L_{2}$) is equal to a quarter wavelength (λ/4).

(7) $$Z_{2}=\sqrt{Z_{1}Z_{3}}$$

The calculated impedance of the quarter-wave section is $Z_{2}$ = 70.7 Ω. The optimized values of the characteristic impedances of the quarter-wave transformer sub-circuit $Z_{1}$ = 100 Ω, $Z_{2}$ = 70.7 Ω, and $Z_{3}$ = 50 Ω are $W_{1}$ = 0.57 mm, $W_{2}$ = 1.41 mm, and $W_{3}$ = 2.78 mm, respectively. The quarter-wave transformer length $L_{2}(\lambda /4$ at frequency 5.25 GHz) is 6 mm.

**Figure 10:**
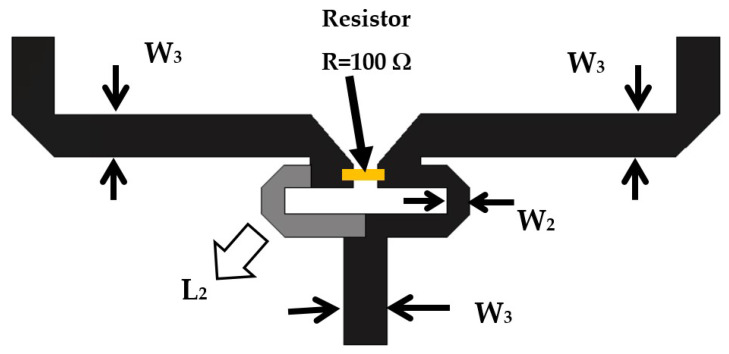
The designed layout of the Wilkinson power divider.

The last element in the feeding circuit is the Wilkinson power divider. Figure 10 shows the designed layout of the Wilkinson power divider. The Wilkinson is designed to divide the power level into two equal power ratios with high isolation at the output port. This high isolation is required to isolate the output terminals of the feeding circuit.

The Wilkinson power divider circuit is designed on FR-4 substrate material with $\epsilon_{r}$ = 4.5, thickness h =1.5 mm, and loss tangent of 0.025. As shown in Figure 10, the input 50-Ω microstrip line is divided into two quarter-wave length microstrip line $(L_{2}\left(\lambda /4\right)$ at frequency 5.25 GHz) with impedance of $Z_{2}$ = 70.7 Ω. The widths of the input/output impedances $Z_{3}$ = 50 Ω and $Z_{2}$ = 70.7 Ω transmission lines are $W_{3}$ = 2.78 mm and $W_{2}$ = 1.41 mm, respectively. The value of the resistor (*R*) is equal to 100 Ω. The length of the quarter-wave length microstrip line $(L_{2}\left(\lambda /4\right)$ at frequency 5.25 GHz) is 6 mm.

**Figure 11:**
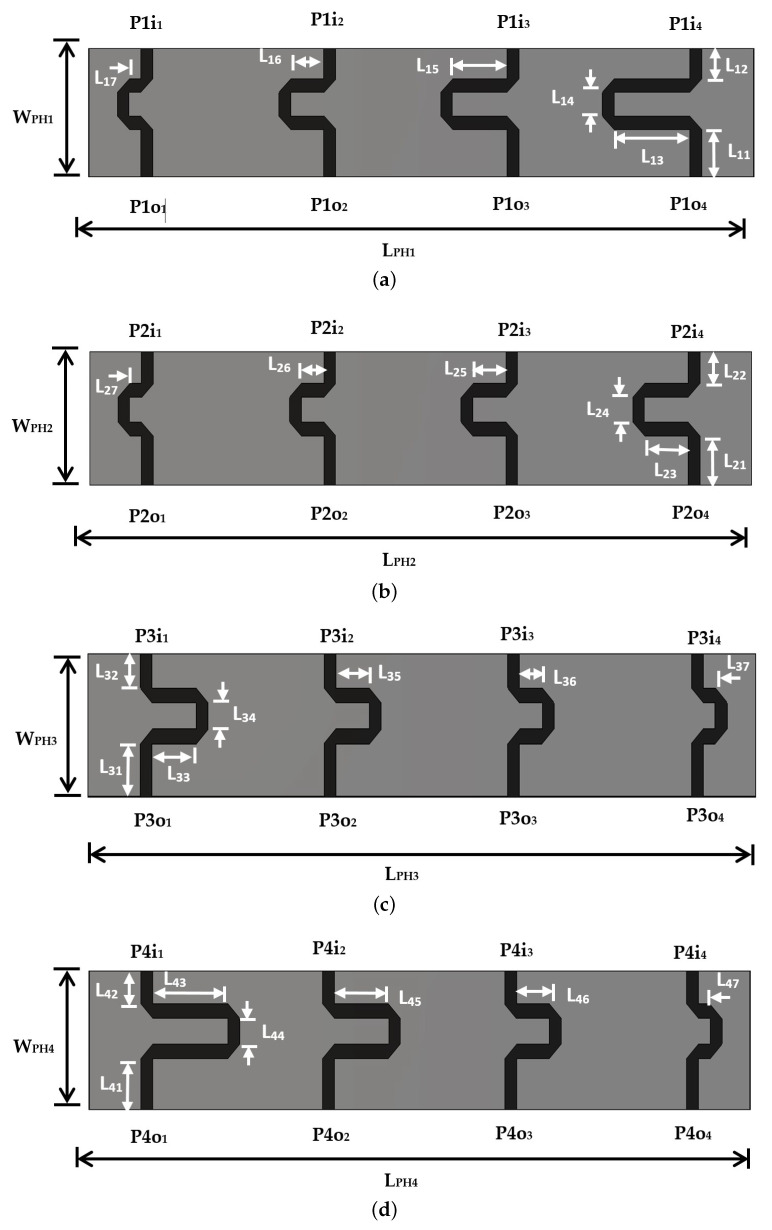
The printed layout configuration of the four extra-phase circuits: (**a**) Extra-phase circuit for inclination angle of 25${}^{\circ }$ toward the right side of Y-axis; (**b**) Extra-phase circuit for inclination angle of 12${}^{\circ }$ toward the right side of Y-axis; (**c**) Extra-phase circuit for inclination angle of 12${}^{\circ }$ toward the left side of Y-axis; and (**d**) Extra-phase circuit for inclination angle of 25${}^{\circ }$ toward the left side of Y-axis.

The extra-phase circuit is designed to obtain an additional phase at the input of the linear antenna array instead of using phase shifters. The proposed extra-phase circuit is composed of four parallel microstrip lines with different lengths. These parallel lines connect the RF signal from the feeding circuit to the linear antenna array with the same magnitude and with different phases. Therefore, an inclined beam of the radiation pattern will occur. As the hall is divided into four horizontal rows and four vertical columns, four different extra-phase circuits are proposed to obtain four different inclined radiation beams. Figure 11 shows the printed layout configuration of the four extra-phase circuits. Table 3 depicts the optimum values for the proposed four extra-phase circuits. All the extra-phase circuits have the same size of $L_{PH}$ = 156.33 mm and $W_{PH}$ = 22 mm. The width of all extra-phase lines is $W_{3}$ = 2.78 mm which represents the impedance of $Z_{3}$= 50 Ω.

Figure 11a,b depict the extra-phase circuits with additional lines where the differences in length of +10 mm and +5 mm are designed to result in two inclined beams at the right side of *Y*-axis direction with inclined angles of 25${}^{\circ }$ and 12${}^{\circ }$ (from broadside), respectively. Figure 11c,d depict another extra-phase circuits with additional lines where the differences in length of −5 mm and −10 mm are designed to result in two different inclined beams at the left side of *Y*-axis direction with inclined angles of 12${}^{\circ }$ and 25${}^{\circ }$ (from broadside), respectively.

### 3.4. The Full Antenna Assembly System

**Figure 12:**
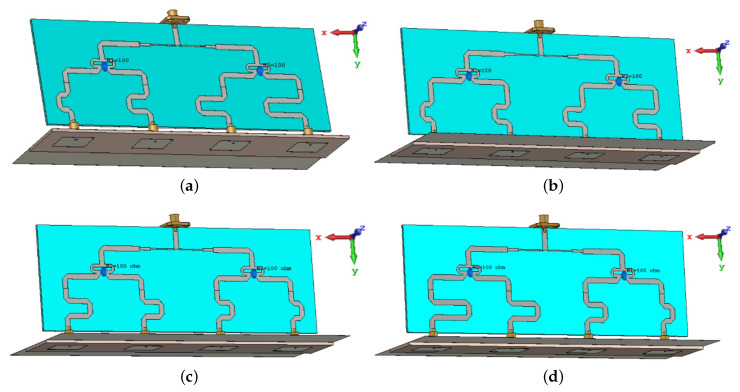
The four RF-sub system: antenna array, feeding circuits and extra-phase circuits, (**a**) RF-circuit for beam inclination of 25${}^{\circ }$ (at right side), (**b**) RF-circuit for beam inclination of 12${}^{\circ }$ (at right side), (**c**) RF-circuit for beam inclination of 12${}^{\circ }$ (at left side), and (**d**) RF-circuit for beam inclination of 25${}^{\circ }$ (at left side).

**Figure 13:**
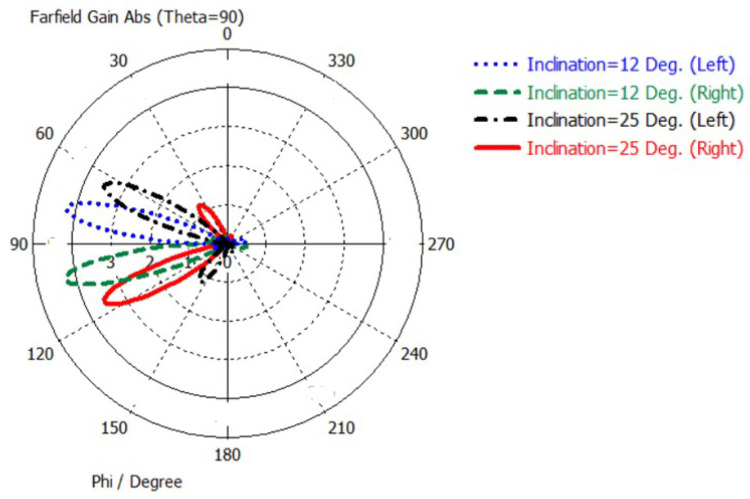
The far-field radiation pattern of the four inclined cases.

Figure 12 shows the four RF-sub system that includes antenna array, feeding circuits, and extra-phase circuits. The simulation is performed using CST Studio Suite software. Figure 13 shows the far-field radiation pattern of the four inclined cases. It is depicted that there are four different inclined beams used to serve four different rows or columns. It is to be noted that the antenna arrays are only parts of the entire access point overall structure. This structure is new, and it is based on switching extra phase circuits to satisfy the orthogonal sweeps. Moreover, in the proposed design, the reflector shape is modified from two inclined reflectors at the circuit side that is in [17], with flat reflector ones. This modification is required for fixing the antenna array assembly within the access point on the ceiling. In addition, the proposed feeding network circuit is different from the two published ones [16,17]. The proposed feeding circuit consists of one T-shaped microstrip feeding circuit and two identical Wilkinson power divider circuits. The feeding circuit that is in [16,17] depends on the T-shaped feeding circuit technique only. Placing two Wilkinson dividers in the proposed structure rejects any received RF signal coming from the antenna array. Therefore, the proposed system introduces an improvement in the system reliability over the published one in [17]. Table 4 shows the angles of the inclined beams versus the values of the extra-phase lines.

## 4. The New AP Automated Design

**Figure 14:**
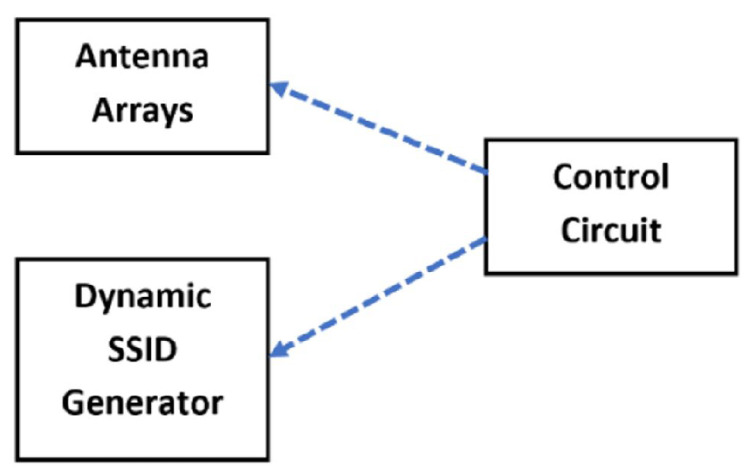
Overall automated AP block diagram.

In an attempt to automate the proposed IPS system, several ideas are investigated. The issue here is to automate the sweeping process from a row (in the horizontal *X* direction) to next and from a column (in the vertical *Y* direction) to next and then start over from the first row and column. This is shown in Figure 14. The first thought is devoted to phase shifters as indicated in [16]. However, after an extensive search, the available phase shifters cover wide ranges of frequency and, as such, the cost is prohibitive to embrace this possibility. The second thought is devoted to using switched stubs with pin diodes; while this thought seems to be attractive, the work is expected to be longer. The adopted thought is focused on the same idea presented in the previous section as the knowledge is there. This is through the use of the extra-phase circuits but now switched automatically.

**Figure 15:**
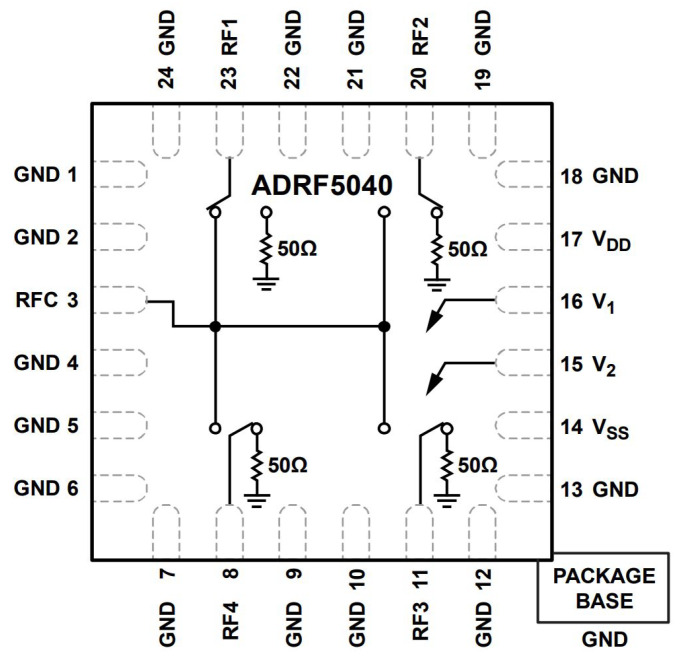
The radio frequency switch.

For the designing of a self-standing AP with the control of the phases, as well as the associated SSIDs, a Raspberry Pi is used along with the following units:

- A USB powered hub to provide enough power to the overall circuitry;
- Two Wi-Fi modules, one to associate with the row covering and the other to associate with the column covering;
- Two 1-to-4 RF switches to switch the extra-phase circuits for sweeping the two antenna arrays to move the footprint coverage from a row (or a column) to the next. This is to be in synchronism with changing SSID of the AP. The chosen RF switch is ADRF5040 by Analog Devices [22]. Its pin layout is shown in Figure 15;
- Two 4 extra-phase circuits as indicated in the previous section and [16];
- Two 4 combiners to combine to each antenna array elements.

**Figure 16:**
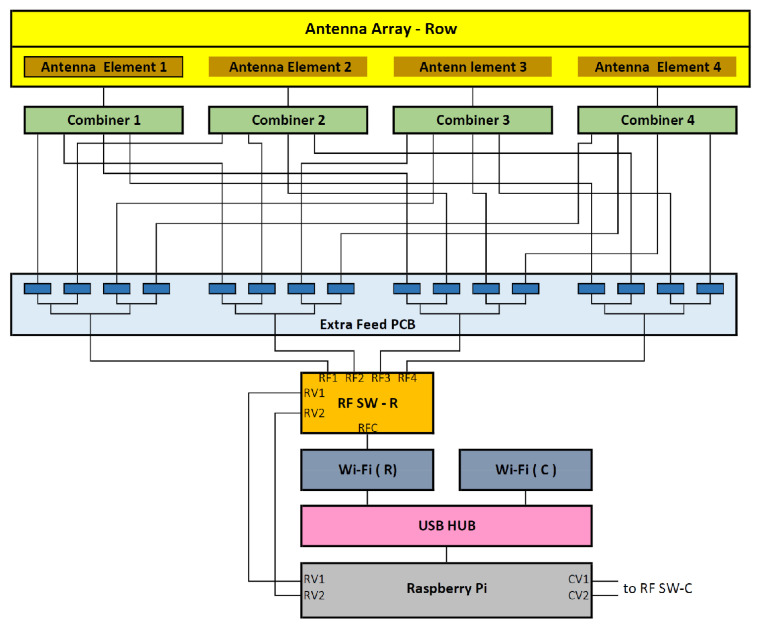
Detailed Design Showing only the Row Schematics.

The schematic block diagram is shown in Figure 16, depicting only the row schematics. Now, the Raspberry Pi (RPi) is the brain of the AP system. The sweeping cycle for the entire hall, as discussed earlier, is shown in Table 5. There are several possible ways of timing control. Here, the rows are swept first, then the columns.

## 5. Discussions and Future Perspective

As mentioned earlier, this paper is a continuation of a research stream based on a new IPS technique. The evaluation design here is a second step from a recently published conference paper, with the objective to have a complete AP design for conducting further research. The overall target is to provide a complete AP design for the location determination, as well as the normal wireless networking objectives. Several research directions can evolve from the concept provided. The next step is to implement the design presented here and then perform unit and system performance testing.

## 6. Conclusions and Future Work

This paper provides a complete access point design capable of performing indoor localization. The positioning technique allows the client to obtain the location coordinates with no overhead. The design consists of a Raspberry Pi as a microcomputer control block, a powered USB hub, two Wi-Fi modules, two RF switches in addition to two antenna array assemblies. The detailed design of the antenna array assembly is given. The overall system operation and block diagram are provided. The implementation, unit testing, as well as overall system performance testing, are in progress.

## Figures and Tables

**Table 1:** Optimum values for the designed rectangular antenna dimensions.

Dimension	Value (mm)
$L_{g}$	25.92
$W_{g}=W_{R}$	34.44
$L_{R}$	20
$L_{p}$	12.55
$W_{p}$	17.22
$X_{p}$	2.89

**Table 2:** Different values of gain for different values of reflector length, with the optimum case in bold.

$L_{R}$ (mm)	Gain (dBi)
No Reflector	11.2
5	11.3
**10**	**11.5**
15	11.2
20	11.1

**Table 3:** The optimum values for the four extra-phase circuits.

Extra-Phase Circuits Dimensions (mm)				
	$\Theta_{1}$ = 25${}^{\circ }$ (toward right side of Y-axis) as in Figure 11a, *i* = 1	$\Theta_{2}$ = 12${}^{\circ }$ (toward right side of Y-axis) as in Figure 11b, *i* = 2	$\Theta_{2}$ = 12${}^{\circ }$ (toward left side of Y-axis) as in Figure 11c, *i* = 3	$\Theta_{2}$ = 25${}^{\circ }$ (toward left side of Y-axis) as in Figure 11d, *i* = 4
$L_{i1}$	6.44	6.44	6.44	6.44
$L_{i2}$	5	5	5	5
$L_{i3}$	17.8	10.3	2.8	2.8
$L_{i4}$	5	5	5	5
$L_{i5}$	12.8	7.8	5.3	7.8
$L_{i6}$	7.8	5.3	7.8	12.8
$L_{i7}$	2.8	2.8	10.3	17.8

**Table 4:** The values of the inclined angles with different extra-phase circuits.

Extra-Phase Length (mm)	Angles of Inclination (from Broad Side)	
	Angle Value (Degree)	Inclination Direction
+10	25	right side
+5	12	right side
−5	12	left side
−10	25	left side

**Table 5:** System sweep cycle.

Phase-Row(X)				Phase-Column(Y)			
Disable Wi-Fi(Y)				Disable Wi-Fi(X)			
Enable Wi-Fi(X)				Enable Wi-Fi(Y)			
T-Row-1	T-Row-2	T-Row-3	T-Row-4	T-Column-1	T-Column-2	T-Column-3	T-Column-3
RF SW-X V1	RF SW-X V1	RF SW-X V1	RF SW-X V1	RF SW-Y V1	RF SW-Y V1	RF SW-Y V1	RF SW-Y V1
V2=LL	V2=LH	V2=HH	V2=HL	V2=LL	V2=LH	V2=HH	V2=HL
Wi-Fi(X) <-	Wi-Fi(X) <-	Wi-Fi(X) <-	Wi-Fi(X) <-	Wi-Fi(Y) <-	Wi-Fi(Y) <-	Wi-Fi(Y) <-	Wi-Fi(Y) <-
SSID-X1	SSID-X2	SSID-X3	SSID-X1	SSID-Y1	SSID-Y2	SSID-Y3	SSID-Y1

## Data Availability

Not applicable.
